# Association of *SCNN1A* Single Nucleotide Polymorphisms with neonatal respiratory distress syndrome

**DOI:** 10.1038/srep17317

**Published:** 2015-11-27

**Authors:** Wang Li, Chen Long, Li Renjun, Hu Zhangxue, Hu Yin, Li Wanwei, Ma Juan, Shi Yuan

**Affiliations:** 1 Daping Hospital, Institute Research of Surgery, Third Military Medical University, Chongqing, China

## Abstract

Increasing evidence has demonstrated that lung fluid absorption disorders might be an important cause of neonatal respiratory distress syndrome (RDS) by influencing gas exchange or surfactant function. The *SCNN1A* gene, which encodes the α-ENaC, might predispose infants to RDS. To explore whether the single-nucleotide polymorphisms (SNPs) of *SCNN1A* are associated with RDS, we conducted a case-control study to investigate the RDS-associated loci in Han Chinese infants. Seven target SNPs were selected from the *SCNN1A* gene and were genotyped using the improved multiplex ligase detection reaction (iMLDR). In the total sample, only rs4149570 was associated with NRDS; this association was further confirmed in logistic regression analysis after adjusting for birth weight, gestational age and sex. In the subgroup of infants whose gestational age was 37 weeks and older, in addition to rs4149570, rs7956915 also showed a significant association with RDS. Interestingly, these associations were only observed in term infants. No significant association was observed between the target SNPs and the risk of RDS in preterm infants. We report for the first time that the rs4149570 and rs7956915 polymorphisms of *SCNN1A* might play important roles in the susceptibility to RDS, particularly in term infants.

Respiratory distress syndrome (RDS) is one of the most important causes of morbidity and mortality in newborns, particularly in those infants born prematurely^1^,^2^,^3^. It has been thought that the etiology of RDS was related to the developmental immaturity of the lungs, particularly of the surfactant synthesizing system. However, recent studies have suggested that^4^,^5^,^6^ the incidence of near-term and term infants with RDS has increased greatly and that their clinical characteristics differ from those of premature infants with RDS. The etiology, pathogenesis and methods of preventing and treating near-term and term infants with RDS have attracted increased attention.

In previous studies, we found that death is inevitable for some babies, despite intensive care and surfactant replacement therapy, particularly in near-term and term infants. Lung tissue slices taken during autopsies of near-term and term infants who died of neonatal respiratory distress syndrome (RDS) showed that, in addition to alveolar collapse from a lack of surfactant, some alveoli were obviously dilated, with a large amount of lung fluid. These findings align with those of previous studies^7^,^8^,^9^ that suggested that lung fluid absorption disorders might be an important cause of RDS by influencing gas exchange or surfactant function, particularly in near-term and term infants.

In our clinical work, we found that some near-term and term infants with RDS that developed from lung fluid absorption disorders showed no obvious signs of infection; furthermore, their mothers did not have any possible risk factors for RDS during pregnancy. Despite antenatal steroid administration, postnatal surfactant therapy, and optimal ventilator care, not all infants of the same gestational age respond equally to treatment. We speculate that this variation may be genetic. Recent clinical observational studies and animal experiments have also shown that there is a close relationship between the risk of RDS and genetic background^10^,^11^,^12^; different populations showed variations in susceptibility to, severity of and treatment response to RDS. Gene polymorphisms are an important material basis for changes in the expression and activity of alpha epithelial sodium channels (α-ENaCs). Because of the influence of traditional etiology, current studies of the genetics of RDS are mainly confined to the gene polymorphisms of pulmonary surfactant protein^13^,^14^,^15^,^16^. To date, there no studies have reported a correlation between RDS and gene polymorphisms of α-ENaCs. In this study, therefore, we selected and genotyped 7 target single-nucleotide polymorphisms (SNPs) within the *SCNN1A* gene to assess the association between α-ENaC and RDS in a Chinese cohort.

## Materials and Methods

### Participants

The control blood samples, which were collected from neonatal umbilical cord blood, were obtained from the maternity ward of 80 hospitals in Chongqing and nearby areas from January 2012 to December 2014. We recruited 171 newborns delivering vaginally or by elective CD, excluding those with RDS (n = 9), neonatal pneumonia (n = 18), meconium aspiration syndrome (n = 5), or other respiratory diseases (n = 10).

Newborns with RDS were consecutively recruited for this study from the neonatal intensive care unit (NICU) at Daping Hospital, Third Military Medical University, Chongqing, China, a tertiary care facility from January 2012 to December 2014. 162 newborns with RDS were recuited for this study, 42 of whom did not complete the study because of death before sampling (n = 16), referral to another hospital (n = 1), parental refusal (n = 11), and defective sampling (n = 14).

The RDS diagnosis was based on clinical manifestations and chest X-ray findings^17^. The clinical signs and symptoms of RDS were progressive respiratory distress, tachypnea, nasal flaring, groan, and cyanosis within 12 hours after birth. The typical X-ray picture of RDS showed a grainy shadow, air bronchogram, and white lung. There were 4 grades, as follows: Grade 1, a slight reticular (slightly granular) decrease in lung transparency, with no obvious difference from normal findings; Grade 2, a soft decrease in lung transparency, with an air bronchogram that overlaps the heart; Grade 3, a gradually stronger decrease in transparency and a blurry diaphragm and heart; and Grade 4, practically homogenous lung opacity^18^. The radiographs were evaluated by two radiologists who were blinded to the patients’ conditions. The infants were excluded if they had any congenital malformation, inherited metabolic abnormalities, intrauterine infection, Rh/Rh incompatibility, pneumonia, pulmonary hypertension, meconium aspiration syndrome, asphyxia, or transient tachypnea of newborns.

Lastly, 249 neonates were eligible for study enrollment (n = 129 without RDS and n = 120 with RDS). Baseline characteristics (gestational age, birth weight and sex) were collected from all infants.

Each participant’s legal representatives gave informed consent for the study, which was approved by the Ethics Committee of Daping Hospital, Third Military Medical University.

## Methods

All the experiments described here were performed in accordance with the regulations issued by the Ethics Committee of Daping Hospital, Third Military Medical University.

### Single nucleotide polymorphism selection and genotyping

*SCNN1A* is located on chromosome 12. The genetic variation data of the entire gene were obtained from the HapMap project (http://hapmap.ncbi.nlm.nih.gov/) for 45 unrelated Chinese Han individuals in Beijing (CHB). Thirty-two SNPs with a minor allele frequency (MAF) of 0.05 or more were identified. Then, we applied Haploview software version 4.2^9^ to choose tag SNPs, which enabled us to capture all common SNPs within the entire SCNN1A gene sequence, according to *r*^*2*^linkage disequilibrium (LD)(threshold ≥0.8). The thirty-two SNPs formed six LD blocks (see Supplementary Fig. S2 online). Nineteen tag SNPs were selected by Haploview software. According to a previous study^19^, seven SNPs were included in this analysis.

Venous blood was sampled into sterile anticoagulation tubes. The genomic DNA was extracted using a Wizard Genomic DNA Purification Kit (Promega, Madison, Wisconsin, USA), according to the manufacturer’s instructions. SNPs were genotyped using the improved multiplex ligase detection reaction (iMLDR), with technical support from the Shanghai Genesky Biotechnology Company.

### Statistical analysis

The birth weights and gestational ages between RDS and control group were compared using a *t*-test. The proportion of females was analyzed using the x^2^ test. Goodness-of-fit to the Hardy-Weinberg equilibrium (HWE) and genotype and allele distributions between RDS and controls were also compared by x^2^ test. Codominant, dominant, recessive, and additive genetic models were applied for genotype distribution analysis. The strength of association between SNPs and RDS was estimated with the odds ratio (OR) with 95% confidence intervals (CIs) by logistic regression, adjusting for birth weight, sex, and gestational age. Statistical analysis was performed using SPSS 16.0 software (SPSS Inc; Chicago, IL). The *P* value for each SNP was corrected by the method of Bonferroni (based on the number of SNPs analyzed). The statistical power of the case-control dataset was evaluated using Power and Sample Size software version 3.0 (http://biostat.mc.vanderbilt.edu/wiki/Main/PowerSampleSize). All of the statistical tests were two-side, with statistical significance set at 0.05.

## Results

### Characteristics of the study population

A total of 249 DNA samples were genotyped, including 120 from the infants with RDS and 129 from the controls. The RDS group had a lower average gestational age (35.03 ± 3.57 vs 36.31 ± 3.10, *P* = 0.003) and birth weight (2.35 ± 0.76 vs 2.60 ± 0.72, *P* = 0.008) compared with the control group. There were no significant differences in sex (female proportion, 0.41 vs 0.45, *P* = 0.485) between the NRDS and control groups. In the RDS group, 113 infants recovered. 35 infants received surfactant more than once. Repeated surfactant rate in three different gestational age stages were 46% (GA ≥ 37weeks), 35% (35weeks ≤ GA<37weeks), and 13% (GA<35weeks). 7 newborns died despite intensive care and surfactant replacement therapy, all of them received surfactant more than once and four of them were near term or term infants.

### Allele frequencies and genotype distribution of target single nucleotide polymorphisms

Detailed information regarding these SNPs including their genome and gene locations, allele frequencies, and p-values for the Hardy-Weinberg equilibrium (HWE) test is presented in Table 1. The MAF of the SNPs in control group were quite similar to the data from the HapMap database. Genotype distributions of the SNPs in the control group were all in agreement with HWE (P > 0.05).

### Single nucleotide polymorphisms of the SCNN1A gene and the risk of neonatal respiratory distress syndrome

In the total sample (Table 2), only the genotype and allele frequencies of rs4149570 were significantly different between the RDS group and the control group (genotype, p = 0.034; allele, p = 0.046). Because the gestational ages and birth weights did not match between the RDS group and the control group, to rule out confounding effects in our initial association analyses, we reevaluated SNP effects under different models using logistic regression adjusting for gestational age, sex and birth weight. Similarly, multivariate logistic regression still revealed that only rs4149570 polymorphism was associated with RDS (additive model: OR = 1.500, 95% CI, = 1.026–2.193, *P* = 0.037; recessive model: OR = 2.386, 95% CI = 1.230–4.629, *P* = 0.010). However, the dominant model did not show a significant association between the rs4149570 polymorphism and the risk of RDS.

Furthermore, we divided these data into 3 subgroups based on gestational age: (a) gestational age ≥37 weeks; (b) 35 weeks ≤gestational age <37 weeks; and (c) gestational age <35 weeks.

In the subgroup of infants whose gestational age was 37 weeks or greater, when genotypes were compared between the RDS and control groups, in addition to rs4149570, rs7956915 also showed a significant difference (P < 0.05) (Table 3).

No significant differences were found for any of the SNPs between the RDS and control groups for the subgroup of infants whose gestational age was ≥35 weeks, <37 weeks and <35 weeks (P>0.05) (see Supplementary Table S1 and Supplementary Table S2 online).

In the total sample, according to the severity of RDS, we divided the infants with RDS into 4 groups, and no significant association was observed between the positive loci (rs4149570 and rs7956915) and the severity of RDS (P>0.05) (see Supplementary Table S3 online).

## Discussion

In present study, there was a trend toward an increased rate of repeated surfactant administration with increasing gestational age. 7 newborns died despite intensive care and surfactant replacement therapy, all of them received surfactant more than once and four of them were near term or term infants. These results were consistent with our previous findings that the surfactant therapy was not effective for all newborns with RDS. Preterm babies <35 weeks of gestational age had a better response to surfactant treatment than near-term and term babies.

If α-ENaC plays an important role in the pathogenesis of RDS by influencing the activity of pulmonary surfactant and lung liquid absorption in neonates^20^,^21^,^22^,^23^,^24^, then the *SCNN1A* gene that encodes α-ENaC might be an important gene that predisposes neonates to RDS.

In this case-control study, we assessed the relationship between 7 candidate polymorphisms of *SCNN1A* and RDS. To our knowledge, this is the first study to examine the genetic associations with RDS from the perspective of lung absorption.

In present study, in the total sample, only one SNP (rs4149570) of the *SCNN1A* gene was found to have a significant association with RDS. This association was further confirmed by logistic regression analysis after adjusting for birth weight, gestational age and sex. In the four different genetic models that could explain the positive association between SNP (rs4149570) and RDS, only three models (i.e., codominant, recessive, and additive) can explain a significant association between rs4149570 and the risk of RDS. The dominant model does not indicate a significant association between rs4149570 and RDS, likely because the A/A genotype (13.9% in the control group and 27.5% in the RDS neonates) was a risk factor for RDS, and the C/C and C/A genotypes were neither a risk factor nor a protective factor for RDS (Table 2). We checked the genotype of rs4149570 for newborns who died of RDS (n = 7), four of them were AA genotype. All of those newborns received surfactant more than once. These results further supported our previous conclusions that the genotype (AA) of the rs4149570 polymorphism within SCNN1A was associated with a higher risk of RDS.

In the subgroup of infants whose gestational age was 37 weeks or greater, in addition to rs4149570, there was another SNP locus (rs7956915) that showed a significant association with RDS when the genotypes were compared between the RDS and control groups (Table 3). Interestingly, these associations were only observed in the group of term infants, and no significant association was observed between any of the target SNPs and the risk of RDS in the preterm infant group (see Supplementary Table S1 and Supplementary Table S2 online). These results were consistent with our hypothesis and previous studies^25^,^26^,^27^ that indicated that the causes of RDS in term infants might differ from those in preterm infants. α-ENaC might play an important role in the pathogenesis of RDS by influencing lung liquid absorption in term infants with RDS.

In addition, according to the severity of RDS, we divided all the infants with RDS in the total sample into four groups, and examine the association between the positive loci (rs4149570 and rs7956915). However, no significant association has been observed (see Supplementary Table S3 online). Considering that RDS is thought to be a multifactorial and/or multigenic disease^28^, the severity of RDS may be modulated by genetics, environmental or gene-environment interactions, and it may be more complicated than simply a matter of SNPs.

We examined the possible functions of rs7956915 and rs4149570 using ENCODE database. SNP rs7956915 is located at RNA binding domain while SNP rs4149570 is mainly involved in the acetylation and methylation of histones. This suggests these two SNPs may influence the transcription of α-ENaC mRNA and then inhibit lung liquid absorption. These functions may explain why the frequencies of AA genotype in rs4149570 and rs7956915 were significantly higher in the RDS group compared with the control group. Further studies are needed to compare the expression levels of α-ENaC mRNA between RDS and control groups.

One noteworthy limitations of this study is the relatively small sample size, mainly due to the difficulties in sample collection of neonatal respiratory distress syndrome. Based on this sample size, the positive association for SNP locus (rs4149570 and rs7956915) that we observed in this study did not remain significant after Bonferroni correction. However, because Bonferroni correction is extremely strict, the rate of false negative may be increased. In addition, in the present study, at a type I error rate of 0.05, the statistical power to detect a relative risk of RDS compared with the control group for SNPs were all below 80%. This suggests that the negative associations for SNPs in this study do not mean there is not a detectable association present, merely we did not have enough power to detect it. Another limitation of this study is the method of investigating the RDS-associated loci. Considering the small sample size in this project, it might be under power to detect rare variants that associated with RDS by deeper resequencing. Since our research purpose is investigating the association between common variants and RDS, we finally chose SNP genotyping as the most cost-effective way. The third limitation is the selection of SNPs, we did not choose all 19 tag SNPs but eliminated part of the SNPs located in the intron and selected 7 SNPs for the analysis according to a previous study^19^. More tag SNPs need to be included for analysis in further studies. Furthermore, we will validate our findings in further studies with larger sample size using deeper resequencing analysis of *SCNN1A* to explore the contributions of both common variants and rare variants to RDS, and provide a stronger biological link to the risk for RDS.

In conclusion, our study suggests that the rs4149570 and rs7956915 polymorphisms in *SCNN1A* might play important roles in the susceptibility to RDS in Han Chinese infants, particularly in term infants. This result supports the assumption that the causes of RDS in term infants might differ from those in preterm infants, and α-ENaC might play an important role in the pathogenesis of RDS by influencing lung liquid absorption in term infants with RDS. However, we did not find any association between the polymorphisms of the *SCNN1A* gene and the severity of RDS. The functions of positive loci (rs4149570 and rs7956915) in the pathogenesis of RDS require further research.

## Additional Information

**How to cite this article**: Li, W. *et al*. Association of *SCNN1A* single-nucleotide polymorphisms with neonatal respiratory distress syndrome. *Sci. Rep*. **5**, 17317; doi: 10.1038/srep17317 (2015).

## Supplementary Material

Supplementary Figure S1

Supplementary Table S1

Supplementary Table S2

Supplementary Table S3

## Figures and Tables

**Table 1:** Information on Genotyped SNPs of SCNN1A.

Gene	Chr	SNP	Position	Region in Gene	Alleles*	MAF (Hapmap-HCB)	MAF (Control)	HWE (Control)
SCNN1A	12	rs4149570	6451590	3′FLANKING	C:A	0.50	0.42	1.00
		rs7297961	6454297	3′FLANKING	G:A	0.07	0.10	0.87
		rs11064145	6455098	3′FLANKING	G:T	0.22	0.25	0.07
		rs13306613	6464809	intron5	C:T	0.09	0.11	0.68
		rs3782724	6466081	intron4	G:A	0.20	0.19	0.41
		rs7956915	6470260	intron4	G:A	0.34	0.30	0.87
		rs11064153	6488450	5′FLANKING	C:T	0.38	0.37	0.08

^*^Major allele: minor allele.

**Table 2:** Genotype distributions of single nucleotide polymorphisms and analysis of their association with RDS.

SNPs	Genotypes	Group, n(%)			Logistic regression			
		CTRL	RDS	P value	Models	OR value	95%CI P	value
Rs11064145	TT	69(54.8)	70(58.3)	0.520	Codominant^a^	0.983	0.584–1.654	0.888
	GT	52(41.3)	48(40.0)			0.632	0.100–3.985	
	GG	5(4.0)	2(1.7)		Dominant^a^	0.962	0.576–1.608	0.884
					Recessive^a^	0.637	0.102–3.964	0.629
					Additive^a^	0.938	0.585–1.506	0.792
rs11064153	CC	46(35.7)	45(37.8)	0.897	Codominant	0.931	0.554–1.594	0.889
	CT	71(55.4)	62(52.1)			1.154	0.447–2.979	
	TT	12(9.3)	12(10.1)		Dominant	0.959	0.569–1.617	0.875
					Recessive	1.204	0.493–2.939	0.683
					Additive	1.014	0.672–1.529	0.949
rs13306613	CC	102(79.7)	103(85.8)	0.321	Codominant	0.672	0.341–1.327	0.519
	CT	25(19.5)	17(14.2)			0.000	0.000–0.000	
	TT	1(0.8)	0(0)		Dominant	0.646	0.329–1.270	0.205
					Recessive	0.000	0.000–0.000	1.000
					Additive	0.631	0.327–1.217	0.169
rs3782724	AA	83(64.3)	78(65.0)	0.474	Codominant	0.859	0.494–1.494	0.543
	GA	42(32.6)	35(29.2)			1.779	0.488–6.491	
	GG	4(3.1)	7(5.8)		Dominant	0.935	0.663–1.613	0.805
					Recessive	1.878	0.523–6.747	0.334
					Additive	1.034	0.663–1.613	0.884
Rs4149570	CC	36(29.5)	30(25)	0.034	Codominant	1.023	0.557–1.879	0.036^b^
	CA	69(56.6)	57(47.5)			2.423	1.116–5.257	
	AA	17(13.9)	33(27.5)		Dominant	1.295	0.727–2.307	0.380
					Recessive	2.386	1.230–4.629	0.010^c^
					Additive	1.500	1.026–2.193	0.037^d^
Rs7297961	AA	103(81.1)	104(86.7)	0.474	Codominant	0.832	0.395–1.753	0.890
	GA	23(18.1)	15(12.5)			0.000	0.000–0.000	
	GG	1(0.8)	1(0.8)		Dominant	0.897	0.431–1.864	0.770
					Recessive	0.000	0.000–0.000	1.000
					Additive	0.970	0.483–1.950	0.932
Rs7956915	GG	61(47.7)	49(41.2)	0.159	Codominant	1.151	0.668–1.984	0.272
	GA	58(45.3)	53(44.5)			2.110	0.852–5.227	
	AA	9(7.0)	17(14.3)		Dominant	1.288	0.767–2.163	0.339
					Recessive	1.963	0.828–5.227	0.126
					Additive	1.335	0.901–1.978	0.149

Abbreviations: RDS, respiratory distress syndrome; CI, confidence interval; CTRL, control; OR, odds ratio; SNP, single nucleotide polymorphism.

^a^Assuming M represents the major allele and m represents the minor allele, genetic models can be described as follows: codominant: M/m vs. M/M and m/m vs. M/M, two OR values were listed from top to bottom in the corresponding column; dominant: (m/m + M/m) vs. M/M; recessive: m/m vs. (M/M + M/m); additive: additive: m/m and M/m were weighed 2 and 1, respectively, to M/M. All models were adjusted by gestational age, birth weight, and sex. Statistically significant values were defined as *p* ≤ 0.05.

^b^Corrected *P* value for multiple testing by Bonferroni correction is 1.000. (*P* value was multiplied by 28 as a Bonferroni adjustment for the 7 SNPs and 4 genetic models tested).

^c^Bonferroni corrected *P*  =  0.280.

^d^Bonferroni corrected *P* = 1.000.

**Table 3:** Association of SCNN1A polymorphism with RDS in infants whose gestational age ≥37 weeks.

SNP	number	Genotypes,n(%)			P value
rs11064145		TT	GT	GG	
RDS	41	27(65.9)	14(34.1)	0(0)	0.185
Control	58	29(50.0)	27(46.6)	2(3.4)	
rs11064153		CC	CT	TT	
RDS	41	14(34.1)	22(53.7)	5(12.2)	0.927
Control	60	20(33.3)	34(56.7)	6(10.0)	
rs13306613		CC	CT	TT	
RDS	41	37(90.2)	4(9.8)	0(0)	0.324
Control	60	50(83.3)	10(16.7)	0(0)	
Rs3782724		AA	GA	GG	
RDS	41	25(61.0)	13(31.7)	3(7.3)	0.629
Control	60	40(66.7)	18(30.0)	2(3.3)	
rs4149570		CC	CA	AA	
RDS	41	12(29.3)	19(46.3)	10(24.4)	0.023^a^
Control	56	18(32.1)	35(62.5)	3(5.4)	
rs7297961		AA	GA	GG	
RDS	41	38(92.7)	3(7.3)	0(0)	0.422
Control	59	50(84.7)	8(13.6)	1(1.7)	
rs7956915		GG	GA	AA	
RDS	41	14(34.1)	20(48.8)	7(17.1)	0.018^b^
Control	60	26(43.3)	33(55)	1(1.7)	

Statistically significant values were defined as p ≤ 0.05.

^a^Corrected *P* value for multiple testing by Bonferroni correction is 0.161. (*P* value was multiplied by 7 as a Bonferroni adjustment for the 7 SNPs tested.)

^b^Bonferroni corrected *P* = 0.126.
