# Supplementary material for: Association of SCNN1A Single Nucleotide Polymorphisms with neonatal respiratory distress syndrome
Source: Sci Rep. 2015 Nov 27;5:17317. doi: 10.1038/srep17317 (PMC4661423; doi:10.1038/srep17317)

**Association of *SCNN1A* Single Nucleotide Polymorphisms with neonatal respiratory distress syndrome**

Wang Li^1^; Chen Long; Li Renjun; Hu Zhangxue; Hu Yin; Li Wanwei; Ma Juan; Shi Yuan^*^

Supplementary Fig. S1 Linkage disequilibrium structure of the SCNN1A


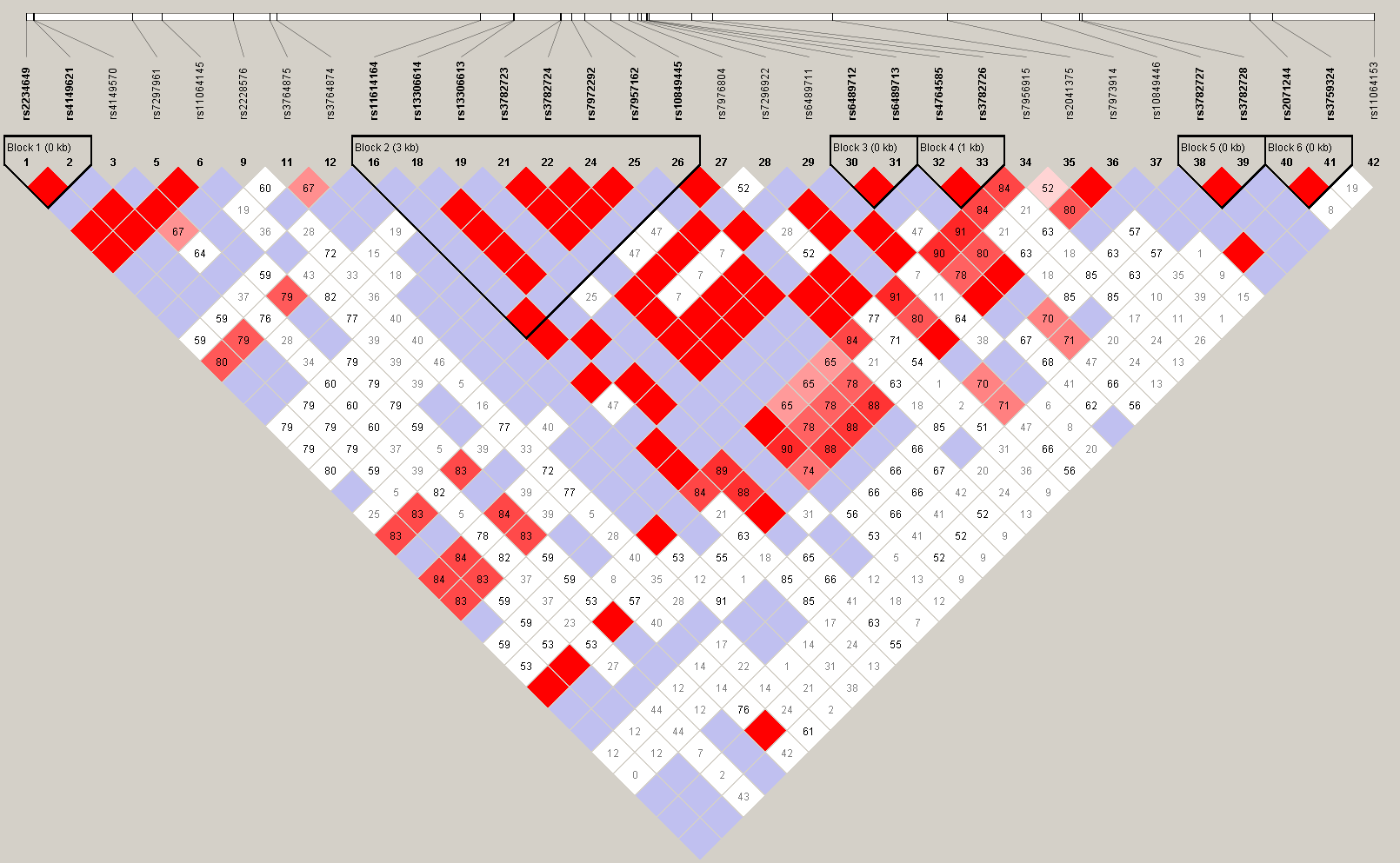

Supplement: Supplementary Figure S1 [file srep17317-s1.doc]
