# Supplementary material for: Association of SCNN1A Single Nucleotide Polymorphisms with neonatal respiratory distress syndrome
Source: Sci Rep. 2015 Nov 27;5:17317. doi: 10.1038/srep17317 (PMC4661423; doi:10.1038/srep17317)
Supplement: Supplementary Table S1 [file srep17317-s2.doc]

**Association of *SCNN1A* Single Nucleotide Polymorphisms with neonatal respiratory distress syndrome**

Wang Li^1^; Chen Long; Li Renjun; Hu Zhangxue; Hu Yin; Li Wanwei; Ma Juan; Shi Yuan^*^

Supplementary Table S1. Association of SCNN1A polymorphism with RDS in infants whose gestational age ≥35 weeks and < 37 weeks.

| SNP | number | Genotypes,n(%) | | | P value |
| --- | --- | --- | --- | --- | --- |
| rs11064145 |  | TT | GT | GG |  |
| RDS | 26 | 14(53.8) | 11(42.3) | 1(3.8) | 0.663 |
| Control | 18 | 11(61.1) | 7(38.9) | 0(0) |  |
| rs11064153 |  | CC | CT | TT |  |
| RDS | 26 | 10(38.5) | 15(57.7) | 1(3.8) | 0.521 |
| Control | 18 | 4(22.2) | 13(72.2) | 1(5.6) |  |
| rs13306613 |  | CC | CT | TT |  |
| RDS | 26 | 20(76.9) | 6(23.1) | 0(0) | 0.429 |
| Control | 18 | 12(66.7) | 5(27.8) | 1(5.6) |  |
| Rs3782724 |  | AA | GA | GG |  |
| RDS | 26 | 18(69.2) | 6(23.1) | 2(7.7) | 0.117 |
| Control | 18 | 9(50.0) | 9(50.0) | 0(0) |  |
| rs4149570 |  | CC | CA | AA |  |
| RDS | 26 | 6(23.1) | 9(34.6) | 11(42.3) | 0.476 |
| Control | 18 | 2(11.1) | 9(50) | 7(38.9) |  |
| rs7297961 |  | AA | GA | GG |  |
| RDS | 26 | 20(77.0) | 5(19.2) | 1(3.8) | 0.276 |
| Control | 18 | 11(61.1) | 7(38.9) | 0(0) |  |
| rs7956915 |  | GG | GA | AA |  |
| RDS | 26 | 10(38.5) | 12(46.2) | 4(15.4) | 0.597 |
| Control | 18 | 8(44.4) | 9(50) | 1(5.6) |  |

Statistically significant values were defined as *p*≤0.05.
