# Supplementary material for: Association of SCNN1A Single Nucleotide Polymorphisms with neonatal respiratory distress syndrome
Source: Sci Rep. 2015 Nov 27;5:17317. doi: 10.1038/srep17317 (PMC4661423; doi:10.1038/srep17317)
Supplement: Supplementary Table S2 [file srep17317-s3.doc]

**Association of *SCNN1A* Single Nucleotide Polymorphisms with neonatal respiratory distress syndrome**

Wang Li^1^; Chen Long; Li Renjun; Hu Zhangxue; Hu Yin; Li Wanwei; Ma Juan; Shi Yuan^*^

Supplementary Table S2. Association of SCNN1A polymorphism with RDS in infants whose gestational age< 35 weeks.

| SNP | number | Genotypes,n(%) | | | P value |
| --- | --- | --- | --- | --- | --- |
| rs11064145 |  | TT | GT | GG |  |
| RDS | 53 | 29(54.7) | 23(43.4) | 1(1.9) | 0.467 |
| Control | 50 | 29(58) | 18(36) | 3(6) |  |
| rs11064153 |  | CC | CT | TT |  |
| RDS | 52 | 21(40.4) | 25(48.1) | 6(11.5) | 0.939 |
| Control | 51 | 22(43.1) | 24(47.1) | 5(9.8) |  |
| rs13306613 |  | CC | CT | TT |  |
| RDS | 53 | 46(86.8) | 7(13.2) | 0(0) | 0.430 |
| Control | 50 | 40(80.0) | 10(20.0) | 0(0) |  |
| Rs3782724 |  | AA | GA | GG |  |
| RDS | 53 | 35(66.0) | 16(30.2) | 2(3.8) | 0.996 |
| Control | 51 | 34(66.7) | 15(29.4) | 2(3.9) |  |
| rs4149570 |  | CC | CA | AA |  |
| RDS | 53 | 12(22.6) | 29(54.7) | 12(22.6) | 0.379 |
| Control | 48 | 16(33.3) | 25(52.1) | 7(14.6) |  |
| rs7297961 |  | AA | GA | GG |  |
| RDS | 53 | 46(86.8) | 7(13.2) | 0(0) | 0.783 |
| Control | 50 | 42(84.0) | 8(16.0) | 0(0) |  |
| rs7956915 |  | GG | GA | AA |  |
| RDS | 52 | 25(48.1) | 21(40.4) | 6(11.5) | 0.673 |
| Control | 50 | 27(54.0) | 16(32.0) | 7(14.0) |  |

Statistically significant values were defined as *p*≤0.05.
