# Supplementary material for: Association of SCNN1A Single Nucleotide Polymorphisms with neonatal respiratory distress syndrome
Source: Sci Rep. 2015 Nov 27;5:17317. doi: 10.1038/srep17317 (PMC4661423; doi:10.1038/srep17317)
Supplement: Supplementary Table S3 [file srep17317-s4.doc]

**Association of *SCNN1A* Single Nucleotide Polymorphisms with neonatal respiratory distress syndrome**

Wang Li^1^; Chen Long; Li Renjun; Hu Zhangxue; Hu Yin; Li Wanwei; Ma Juan; Shi Yuan^*^

Supplementary Table S3. Association of SCNN1A polymorphism(rs4149570, rs7956915) and severity of RDS.

| SNP | Severity of RDS | number | Genotypes,n(%) P value | | | |
| --- | --- | --- | --- | --- | --- | --- |
| rs4149570 |  |  | CC | CA | AA |  |
|  | Ⅰ | 35 | 7(20.0) | 17(48.6) | 11(31.4) | 0.710 |
|  | Ⅱ | 41 | 13(31.7) | 16(39.0) | 12(29.3) |  |
|  | Ⅲ | 26 | 4(15.4) | 15(57.7) | 7(27.0) |  |
|  | Ⅳ | 18 | 5(27.8) | 9(50.0) | 4(22.2) |  |
| rs7956915 |  |  | GG | GA | AA |  |
|  | Ⅰ | 35 | 13(37.1) | 18(51.4) | 4(11.4) | 0.867 |
|  | Ⅱ | 41 | 19(46.3) | 15(36.6) | 7(17.1) |  |
|  | Ⅲ | 26 | 9(34.6) | 13(50.0) | 4(15.4) |  |
|  | Ⅳ | 17 | 8(47.0) | 7(41.2) | 2(11.8) |  |

Statistically significant values were defined as *p*≤0.05.
